# Bladder Paraganglioma

**DOI:** 10.5334/jbsr.2064

**Published:** 2020-05-12

**Authors:** Bert Degrieck, Pieter De Visschere, Bruno Lapauw

**Affiliations:** 1 UZ Ghent, BE

**Keywords:** Bladder MRI, Bladder lesion, Bladder paraganglioma, Bladder mass, Bladder tumor, Paraganglioma, Submucosal lesion

## Abstract

**Teaching Point:** A submucosal bladder wall lesion with high signal on T2-weighted MRI warrants blood and urine analysis to rule out a paraganglioma.

## Case History

**Figure 1:**
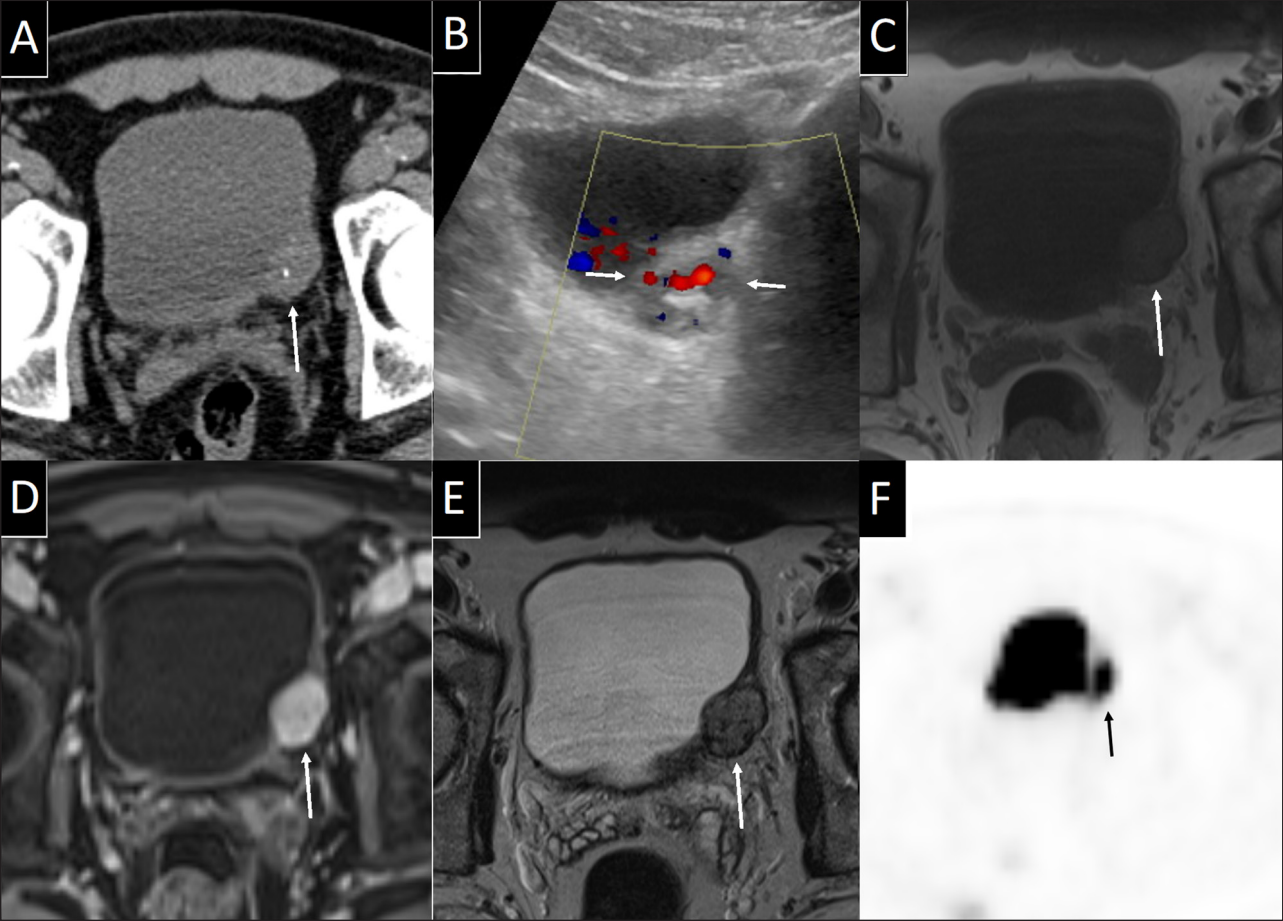
**(A)** Axial non-contrast enhanced CT image showing the incidental ovoid soft tissue mass (white arrow) with a punctate calcification in the left posterolateral bladder wall. **(B)** Doppler ultrasound image showing the lesion (arrows) to have a heterogeneous echogenicity, and marked Doppler signal revealing high vascularity. **(C)** Axial T1-weighted MRI shows the lesion (arrow) to have an intermediate T1 signal and **(D)** axial fat suppressed T1 after i.v. gadolinium shows marked and homogeneous lesion contrast enhancement (arrow). **(E)** Axial T2 showing the lesion (arrow) to be located intramurally, with intact overlying urothelium. and having a heterogeneous moderately high T2 signal. **(F)** Axial F-18 FDG PET image shows moderate FDG uptake of the lesion (arrow). Also note the renally excreted FDG in the bladder lumen.

A 57-year-old male presented with right flank colic pain. Non-contrast computed tomography (CT) (Figure 1A) confirmed right-sided obstructive urolithiasis but also incidentally revealed a sharply delineated oval mass (2.6 cm) in the left bladder wall containing a punctate calcification. Complementary Doppler ultrasound (Figure 1B) showed well-vascularized lesion. Venous phase contrast-enhanced CT (not shown) showed homogeneous and marked enhancement. Additional magnetic resonance imaging (MRI) (Figure 1C–E) located the lesion in the submucosa, with intact overlying urothelium. The lesion had an intermediate signal on T1-weighted images but a moderately high signal on T2-weighted images. Fat saturated T1-weighted images after contrast administration also showed marked and homogeneous contrast enhancement. These imaging findings were not typical for urothelial cell cancer (UCC), but suggestive for a tumor of neuroendocrine, vascular or muscular origins.

Blood and urine work-up for excess catecholamine production showed slightly elevated urine normetanephrine and noradrenaline, suggesting the diagnosis of a paraganglioma. A cystoscopic biopsy specimen was obtained and the pathology results showed a lesion morphologically resembling UCC but expressing synaptophysin, leading to the diagnosis of neuro-endocrine tumour (i.e. paraganglioma). A whole-body 18F-fluoro-deoxyglucose positon emission tomography coupled with CT (F-18 FDG PET-CT) (Figure 1F) showed moderately high uptake of the tracer in the bladder lesion and no distant metastasis. The patient was treated with robot-assisted partial cystectomy after pre-treatment with alpha-blockage. No intra-operative hypertensive crisis occurred. Follow-up is performed by regular blood and urine metanephrine samples and imaging.

## Discussion

Bladder paraganglioma (sometimes also referred to as extra-adrenal pheochromocytoma of the bladder wall) is an exceedingly rare tumour arising from the chromaffin cells of the bladder, which explains their intramural location. They account for 0.05% of bladder tumours and for less than 1% of paragangliomas [1]. On MRI imaging these lesions typically show a high T1 signal and high T2 signal with a “salt and pepper” aspect. The differential diagnosis based on imaging characteristics includes haemangioma, leiomyoma and UCC. Around 10–15% of the bladder paragangliomas are non-functioning and a further 10% have hormonal activities that do not always manifest clinically. This makes it hard to come to the correct diagnosis prior to surgery, which may result in an unexpected intraoperative hypertensive crisis because of possible catecholamine excess produced by these lesions. If paraganglioma is suspected on imaging, blood and urine sample analyses should be carried out.
